# Physical activity cut-points for older adults using the Zio XT onboard accelerometer

**DOI:** 10.1186/s44247-024-00087-8

**Published:** 2024-06-12

**Authors:** Lacey H. Etzkorn, Anis Davoudi, Erin E. Dooley, Kelley P. Gabriel, Lin Yee Chen, Ciprian M. Crainiceanu, Jennifer A. Schrack, Amal A. Wanigatunga

**Affiliations:** 1 Department of Epidemiology, Bloomberg School of Public Health, Johns Hopkins University, Baltimore, MD, USA; 2 Johns Hopkins Center On Aging and Health, Baltimore, MD, USA; 3 Department of Epidemiology, The University of Alabama at Birmingham, Birmingham, AL, USA; 4 Lillehei Heart Institute and Cardiovascular Division, University of Minnesota Medical School, Minneapolis, MN, USA; 5 Department of Biostatistics, Bloomberg School of Public Health, Johns Hopkins University, Baltimore, MD, USA

**Keywords:** Actigraphy, Wearable activity monitors, Electrocardiogram

## Abstract

**Introduction:**

The Zio^®^ XT continuous ambulatory electrocardiographic monitor (Zio) contains an accelerometer that can help quantify an individual’s physical activity in the free-living environment.

**Purpose:**

(1) To estimate activity cut-points to estimate daily time spent performing very light, light, or moderate to vigorous physical activity (VLIPA, LIPA, MVPA) for the Zio accelerometer. (2) To describe how Zio’s 24-h wear protocol affects estimates of daily MVPA relative to a waist-worn accelerometer’s waking-wear protocol.

**Methods:**

Three hundred eighty one participants from the Atherosclerosis Risk in Communities (ARIC) study wore a waist-mounted ActiGraph GT3X (except while sleeping or water-based activities) and a Zio (24-h) simultaneously for three to seven days. For each person-minute, physical activity was summarized as the Mean Amplitude Deviation (MAD) for the Zio and Vector Magnitude Counts (VMC) for the GT3X. Cut points previously used in ARIC were mapped from GT3X VMC to Zio MAD using a conditional two-sample quantile–quantile approach.

**Results:**

Evenson VMC cut-points for the GT3X (VLIPA≥76, LIPA≥903, MVPA≥2075 counts/min) were mapped to MAD for Zio (9.04, 28.2, and 58.1 mili-g). Daily hours spent in each intensity category were each strongly correlated (ICC > 0.7) between summaries produced by Zio and GT3X when restricting estimates to overlapping wear time. Zio and GT3X-estimated MVPA had high agreement (ICC = 0.77) when using device-specific wear time, but Zio measured one hour more of daily LIPA and VLIPA (95% CI = 0.83, 1.07 hrs/day).

**Conclusions:**

We recommend the use of our cut-points for clinical research with Zio accelerometry in populations of older adults.

## Introduction

The Zio XT (iRhythm Technologies, Inc., San Francisco, CA, USA) is a continuous ambulatory electrocardiographic monitor that also contains an onboard accelerometer, which has recently been demonstrated to be a useful tool for measuring physical activity (PA) and sedentary behavior [1–3]. However, methods for quantifying PA with Zio have not been fully developed. Processing accelerometer data for health research requires summarizing high-dimensional, multivariate timeseries data into meaningful summary variables to be used in further analysis. For example, summaries commonly used with the other accelerometers include total activity counts (TAC), which conveys the wearer’s total amount of daily PA [4]. However, many health researchers are unfamiliar with the units and typical distributions of counts, so results can be unintuitive. Another common technique for quantifying PA is to summarize the daily minutes a participant spends at various levels of movement intensity (i.e., light, moderate, and vigorous intensity PA) [5]. Since these summaries have units of daily minutes or hours, they are often more intuitive for end-users, including health researchers and the general population.

To date, daily time spent performing light and moderate to vigorous intensity PA has not been quantified with Zio as this method requires the development of cut-points (i.e., the threshold value of an activity intensity metric which distinguishes two adjacent activity categories). Accelerometer cut-points for Zio cannot be borrowed directly from previous work from other accelerometers because the magnitude and distribution of activity intensity measurement can vary by the model of accelerometer, the body placement of the accelerometer, the metric used to quantify intensity of movement, and the population to which the accelerometers are applied. For example, previous work in Atherosclerosis Risk in Communities study (ARIC) used Evenson [6] cut-points designed for the movement intensity metric vector magnitude of activity counts (VMC), which were derived from the ActiGraph GT3X (ActiGraph LLC, Pensacola, FL, USA) mounted at the waist among older adults (aged 60 years and older) [7]. In contrast, Zio is applied to the chest and contains a different model of accelerometer. Furthermore, VMC is an activity intensity metric developed by ActiGraph for their own accelerometers that was proprietary until recently [8]. Hence, it is unknown whether VMC can be quantified with Zio reliably. Instead, previous studies with Zio accelerometer data have characterized movement intensity via mean amplitude deviation (MAD), an open-source metric of activity intensity [1, 3, 9].

To develop cut-points for Zio, we used data from ARIC participants who wore Zio and the GT3X simultaneously during ARIC visit six (2016–2017). These data allow us to characterize the association between Zio MAD and GT3X VMC measurements of movement intensity and to appropriately map cut-points from the latter device to the former. To examine the validity of our cut-points, we first verify that these Zio MAD cut-points produce similar estimates of daily hours spent in movement intensity categories compared to the GT3X. In a second test of validity, we explore whether our Zio cut-points yield activity estimates with similar associations to a few known, key correlates of PA such as time of day, day of week, sex, age, and body mass index (BMI) [7]. In a secondary analysis, we explore how the 24-h wear protocol for Zio affects the estimates of time spent in each activity category relative to the waking-wear protocol for the GT3X. The creation of Zio cut-points will help to produce interpretable measures of daily PA and thus illuminate associations between health outcomes and objectively-measured PA. Our in-depth comparison of minute-level and day-level physical activity summaries will aid in the harmonization of data and results from studies which use Zio and studies which use the GT3X.

## Methods

### Study design and physical activity assessment

Briefly, between 1987 and 1989, the Atherosclerosis Risk in Communities study (ARIC) enrolled 15,792 adults aged 45 to 64 years from North Carolina, Mississippi, Minnesota, and Maryland to surveil cardiovascular disease and cardiovascular-related deaths in these areas [10]. In the initial phase of cohort surveillance (1987–1998), ARIC participants attended four study visits roughly every three years. To monitor health of ARIC participants in later life, living participants were invited to additional visits five (2011–2013), six (2016–2017), and up to visit ten (2023). Participants who attended visit six were invited to wear Zio. Participants were excluded from participation if they had previously or currently had an implanted cardiac electronic device or allergic reaction to adhesive tape. Participants were instructed to wear Zio twenty-four hours per day for two weeks and return Zio via prepaid mailers to iRhythm for data processing. Of the 4,003 visit six participants, 3,680 were eligible and 2,650 agreed to wear Zio and provided written and informed consent [11].

ARIC participants who attended clinic visit six from May through November of 2016 were also invited to participate in the Physical Activity and Falls Ancillary Study. Participants were excluded if they resided in a total care nursing home, had heightened risk of dementia, were unable to perform the four-meter gait speed assessment, or did not have their visit in the period of May through November of 2016 [12]. Participants were asked to wear the GT3X for seven days on their waist and to remove the device only when going to bed or during water-based activities, such as swimming or bathing (i.e., “waking-wear protocol”). Participants were instructed to return the GT3X to study staff via prepaid mailers. A total of 539 participants were eligible and agreed to wear the device. The ARIC study was approved by the institutional review board at each participating center and informed consent was obtained from all participants.

### Physical activity measurement

GT3X files were downloaded in ActiLife 6 and re-integrated into “.agd” files at 60s epochs. Data were screened for non-wear using the Choi algorithm [13], and any data recorded beyond the sixth day was discarded. Participants were retained for further analysis who had ≥ 600 min of wear time on three or more distinct calendar days. A single additional participant was removed due to a high degree of discordance between the Zio and GT3X data (Appendix A). Minute-level PA from the GT3X was summarized as VMC (denoted as VMC_G_), a metric of activity intensity produced by ActiLife software (ActiGraph LLC, Pensacola, FL, USA) [7]. Zio accelerometer data were screened for non-wear both visually and algorithmically [14], and minute-level PA was summarized as MAD (denoted as MAD_Z_).

### Estimation of the mapping function and cutpoints

To construct a function that maps minute-level VMC_G_ values to equivalent minute-level MAD_Z_ values, we constructed a two-sample quantile–quantile (QQ) function. In a two-sample QQ function, repeated measurements of two random variables (MAD_Z_ and VMC_G_) are sorted into order statistics, and the *i*th order statistic from the distribution of MAD_Z_ is paired with the *i*th order statistic from the distribution of VMC_G_. An interpolation can be drawn between the points on this graph, and the result is a non-decreasing function. (See Tsai [15] for further description of quantile–quantile functions.) The QQ function was constructed by pooling minute-level MAD_Z_ and VMC_G_ measurements from overlapping wear on valid days (i.e., days with ≥ 600 min of wear time).

We chose to use this QQ approach rather than regression of MAD_Z_ on concurrent VMC_G_ measurements as in Karas et al. because the QQ approach does not rely on the clocks in the devices being perfectly synchronized [16]. Instead, the QQ approach only assumes that the underlying distribution of minute-level activity intensities are the same across the population.

For the GT3X, previous ARIC investigations used a version of activity cut-points developed by the Woman’s Health Initiative (WHI) for waist-mounted ActiGraph accelerometers to demarcate inactive (< 76 counts/min), very light (76 to 902 counts/min), light (903 to 2074 counts/min), and moderate to vigorous intensity PA (> 2074 counts/min) [6, 7]. These cut-points were mapped from VMC_G_ to MAD_Z_ using the QQ function. Confidence intervals for the MAD_Z_ cut points were generated using a clustered bootstrap procedure with 1,000 bootstrap replicates. The clustered bootstrap procedure resamples data clusters (i.e., study participants) with replacement rather than units within clusters (i.e., wear minutes) to generate confidence intervals which account for this multi-level data structure [17].

### Summaries of physical activity

Daily time spent performing very light intensity PA (VLIPA), light intensity PA (LIPA), and moderate-to-vigorous PA (MVPA) were estimated from GT3X data for each participant (Appendix A). For the purposes of this work, we define “sleep and sedentary time” (SST) as the daily time not spent in VLIPA, LIPA, or MVPA. Because participants were asked to remove the device for sleeping, showering, and bathing, daily MVPA, LIPA, VLIPA, and SST are estimated by assuming non-wear reflects SST.

To address the two distinct goals of our analysis, Zio summaries were computed in two ways. First to assess the validity of the cut-points (Scenario 1), Zio data were restricted to overlapping wear on valid days, the same as for building the QQ function (Sect. “Estimation of the mapping function and cutpoints”). By restricting GT3X and Zio data to their common wear time before calculating the Zio daily summaries, we are able to remove any additional discrepancy in the estimates due to differences in the wear protocol (24 h vs. waking-wear).

Our second aim is to characterize how Zio summaries and GT3X summaries compare when estimated with respect to their device-specific protocols. Hence, in Scenario 2, we compute Zio summaries for each valid day, but we no longer restrict Zio data to the GT3X wear time. For Scenario 2, a valid day is defined as at least 10 h of GT3X data and 24 h of Zio data. In Scenario 2, we expect that estimated VLIPA, LIPA, and MVPA from the Zio will be higher relative to the same Zio summaries from Scenario 1.

### Statistical analysis

For both Scenarios 1 and 2, sample percentiles of time spent in each activity category were compared between devices, and agreement was assessed via Pearson’s correlations, intra-class correlations (ICC), mean differences, and standard deviations of differences. Confidence intervals for mean differences use the *t* distribution, and confidence intervals for the ICCs are bias-corrected and accelerated bootstrap intervals based on 1,000 bootstrap replicates. Associations of PA summaries with time of day, day of week, sex, race, age, and BMI were visually compared by device (Figs. 2, 3 and 4).

## Results

### Sample characteristics

Zio and GT3X accelerometer files were obtained for 450 ARIC visit 6 participants. Of the 450 participants with Zio data, 381 had at least 3 valid days with at least 10 h of overlapping GT3X and Zio measurements (Scenario 1), and 378 had at least 3 overlapping valid days of GT3X and Zio data (Scenario 2). Under Scenario 1, included participants had a median of 5 days with valid wear (Q1 = 5, Q3 = 5) and a median of 14.0 h of GT3X wear per valid day (Q1 = 13.1, Q3 = 15.0). These ARIC participants had similar proportion of female participants (56.7%) and similar distribution of BMI (median = 27.4 kg/m^2^) compared to the other ARIC visit 6 participants (Table 1). However, ARIC participants in the present analysis had more white individuals (80.6% vs. 74.7%) and had a median age that was one year younger (78 vs. 79 years, *p* < 0.001).

### QQ Mapping and cut-points

**Fig. 1:**
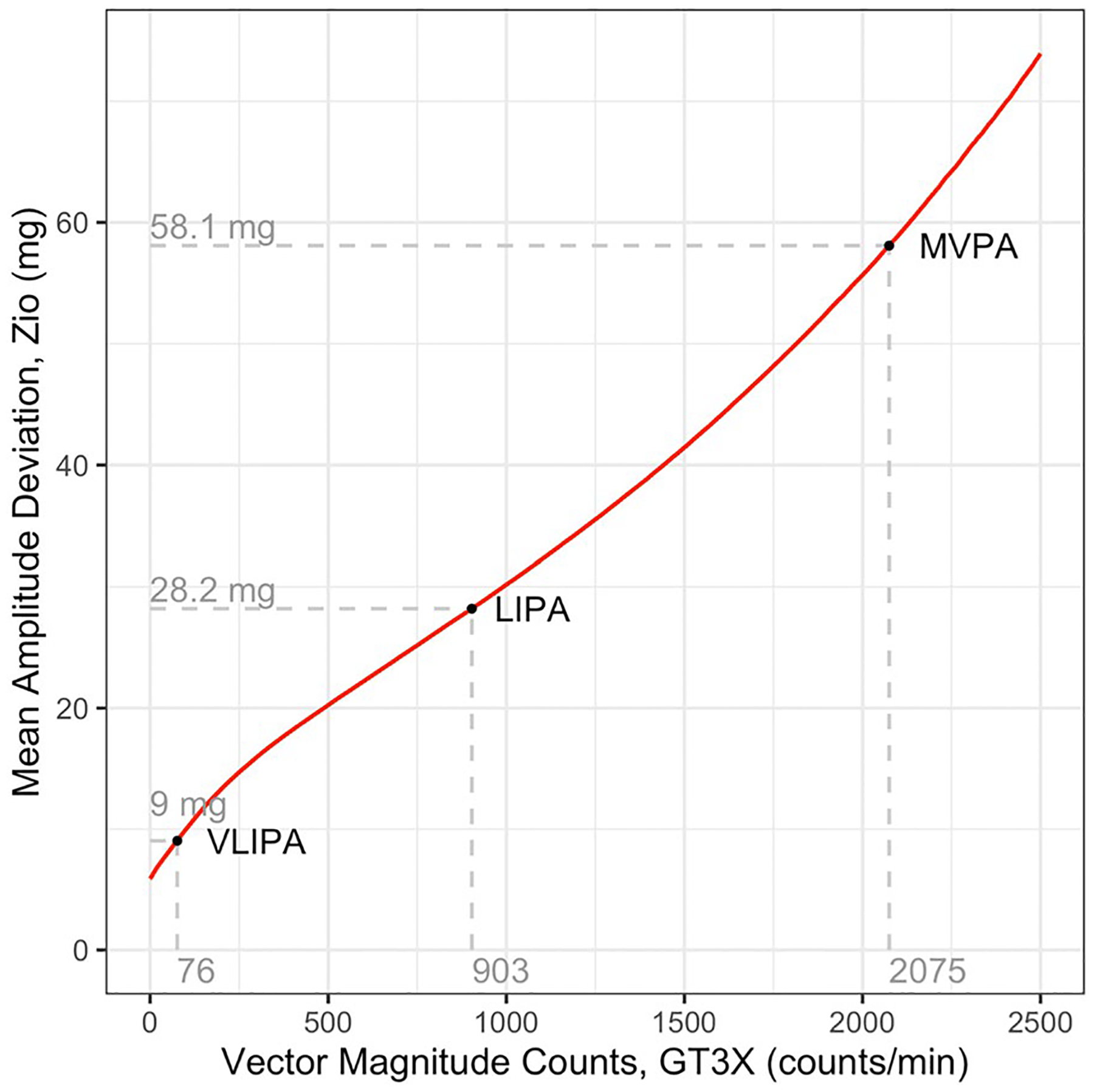
Population quantile–quantile mapping of minute-level mean absolute deviation for Zio XT and Vector Magnitude Counts for Actigraph GT3X, Note: Points marked VLIPA, and LIPA, and MVPA represent the lower-bound cut-points for very light, light, and moderate-to-vigorous intensity categories. Both axes have been windsorized. A tabulation of this mapping is included in Appendix C

Figure 1 displays our estimated transformation equation (i.e., QQ function), which maps minute-level VMC_G_ measurements to minute-level MAD_Z_, and Appendix C provides a tabulation of this mapping. The first column of Table 2 lists cut-points which were previously used in the ARIC study. The second column lists the percentage of valid wear intervals which fell in the given activity category. The third and fourth columns provide QQ mappings of the VMC_G_ cut-points to MAD_Z_ with confidence intervals for those mappings.

### Activity summaries

Table 3 describes distributions of individual-level activity summaries from each device for Scenario 1, which compares summaries based on common wear time. In Scenario 1, there was strong agreement (ICC > 0.7) between devices for very light, light, and moderate-to-vigorous PA. Average daily minutes spent in each activity category had similar means between devices: all confidence intervals for the mean difference included 0 (Table 3).

Table 4 demonstrates that when using the devices’ respective wear protocols (Scenario 2), daily hours of MVPA maintained good agreement between the Zio and GT3X (ICC = 0.765, Mean Difference = 4.9 min/week) relative to Scenario 1 (Table 3: ICC = 0.769, Mean Difference = 0.3 min/week). The average Zio-estimated daily SST was nearly one hour lower than GT3X-estimated SST (95% CI = −1.07, −0.83 h/day). This difference was offset by higher daily Zio-estimated VLIPA (Mean Diff. = 0.77 h/day) and LIPA (Mean Diff. = 0.17 h/day) compared to the GT3X. Though the explicit agreement between devices of estimated SST, VLIPA, and LIPA were slightly weaker in Scenario 2 (ICCs = 0.7, 0.55, 0.68), there were still strong linear associations between estimates by device (Pear. Cor. = 0.81, 0.71, 0.7).

**Fig. 2:**
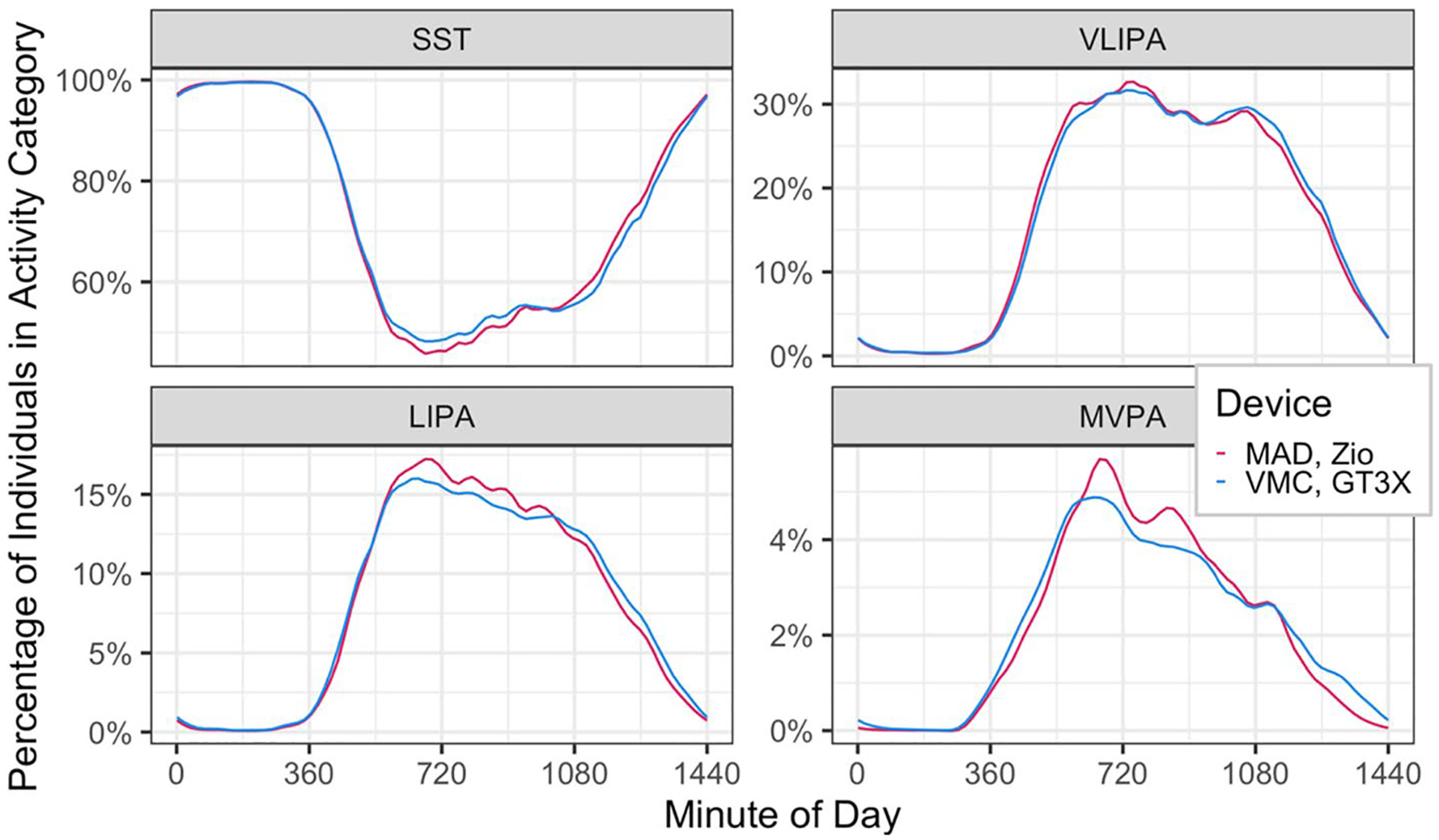
Diurnal patterns of percentage of individuals in activity categories

**Fig. 3:**
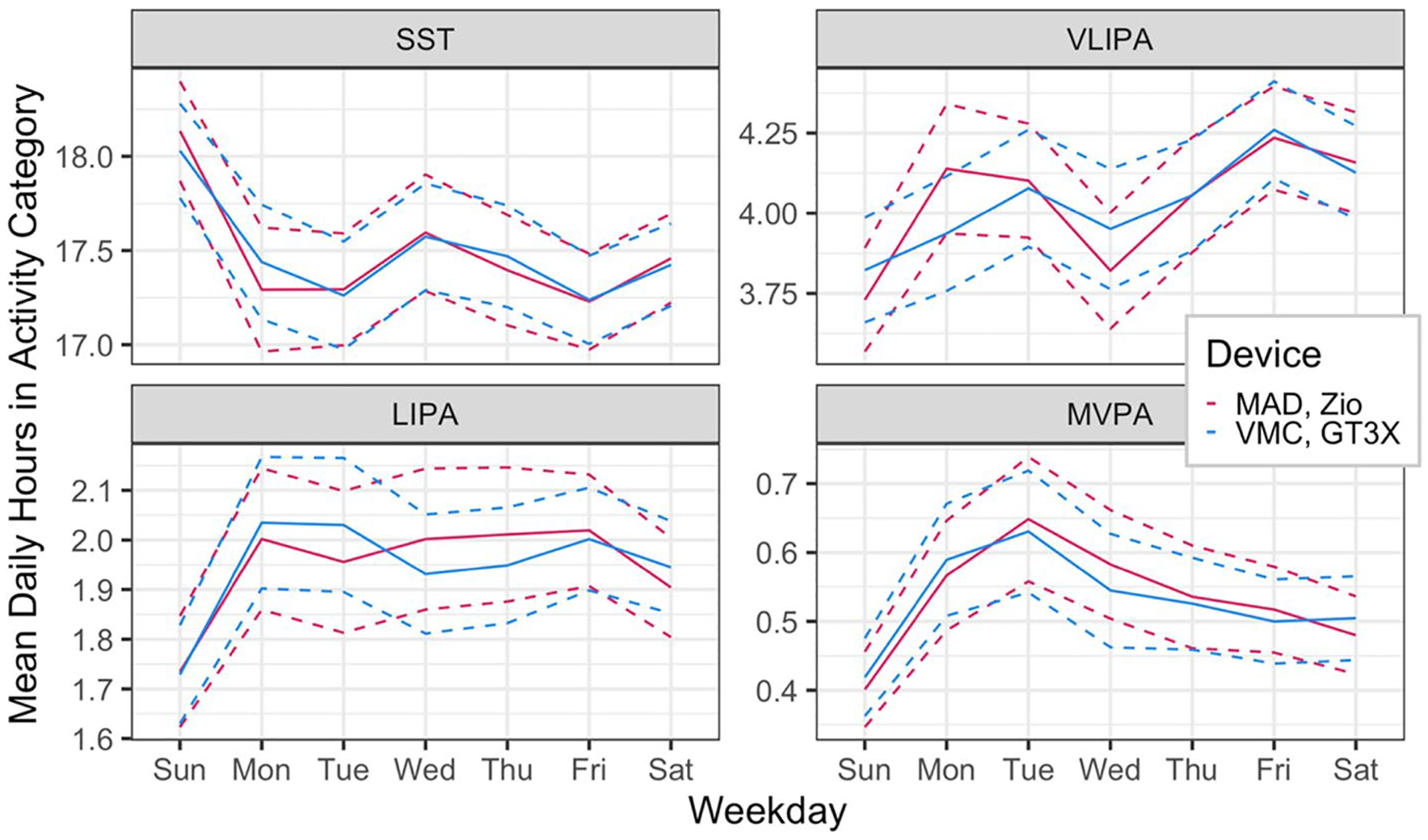
Weekly patterns of daily hours spent in activity categories with confidence intervals

**Fig. 4:**
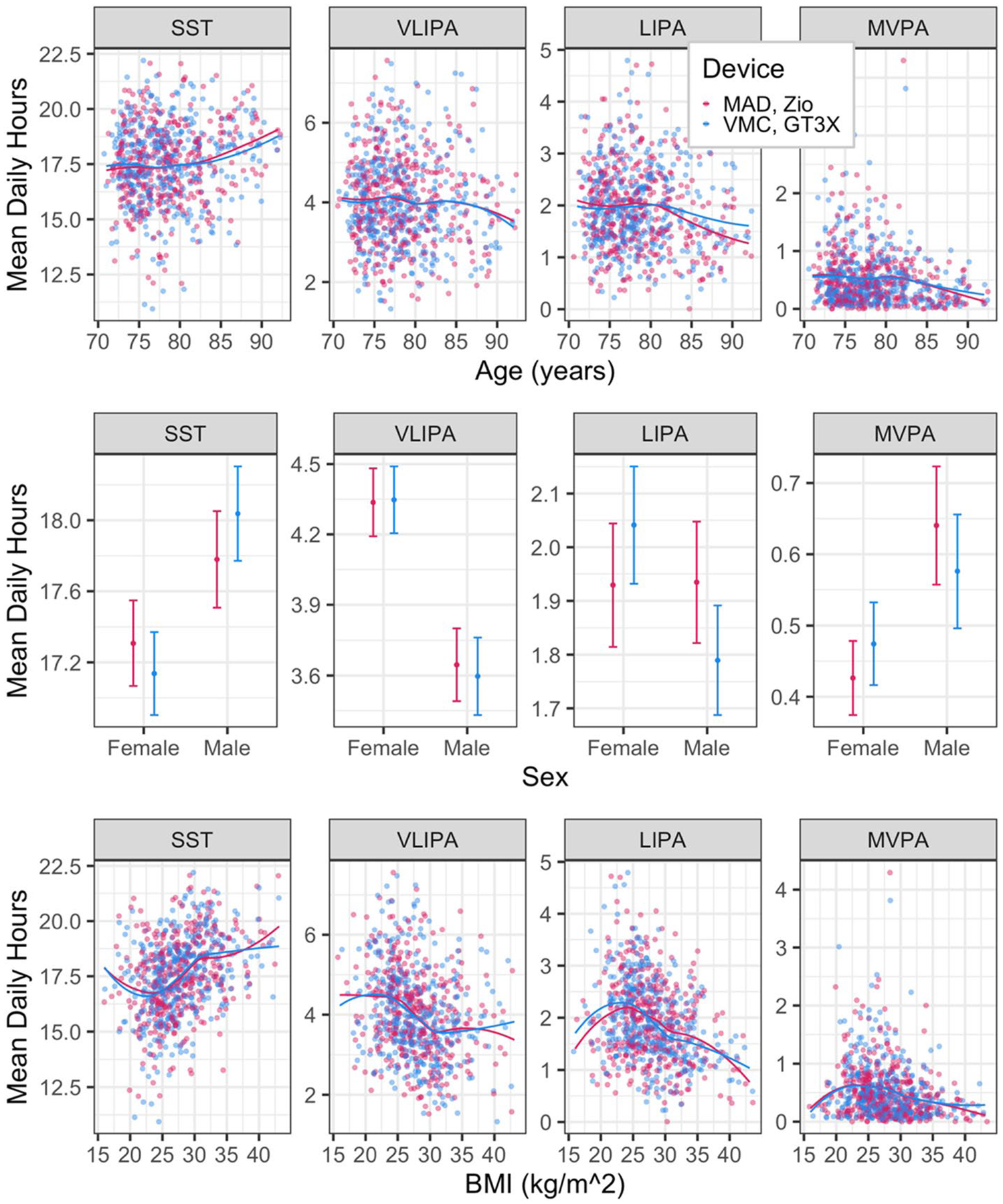
Associations of age, sex, and BMI with mean daily hours spent in activity categories by device

Figure 2 describes the proportion of individuals in each activity category by time of day and device. Similarly, Figs. 3 and 4 describe the association of daily proportion of time spent in each activity category with day of week, gender, age and BMI. The character of the association with all factors, except sex, and activity summaries were similar between devices. Sex-related differences in SST and LIPA were attenuated when measured by Zio compared to the GT3X. Sex-related differences in MVPA were inflated when measured by Zio.

## Discussion

We estimated PA cut-points for the Zio XT onboard accelerometer, and we provided a mapping function for researchers who wish to map other VMC cut-points designed for a waist-mounted ActiGraph GT3X. To validate our cutpoints, we demonstrated that Zio-estimated daily VLIPA, LIPA, and MVPA had high agreement (ICC ≥ 0.7) with estimates from the ActiGraph GT3X, a well-studied and validated accelerometer. As a second test of validity, we showed that Zio PA intensity estimates had similar associations with key participant characteristics (i.e., age, BMI), day of the week, and time of the day. Collectively these results suggest these new cut-points can be used with Zio to characterize daily distribution of PA intensity for older adults in clinical research.

In a second aim, we sought to use our Zio cut points to describe expected practical differences in estimates of daily PA between studies which use Zio, which uses a 24-h wear protocol, and studies which use the waist-worn GT3X with waking-wear protocol. The differences in wear protocol had very little effect on estimated MVPA (ICC = 0.77; mean difference = 4.9 min/week) but had a more substantial effect on estimated VLIPA and LIPA (ICC = 0.55, 0.68). This suggests that studies which use Zio to estimate MVPA should be directly comparable to studies that estimated MVPA with waist-worn devices and a waking-wear protocol. However, those same two studies may not generate comparable estimates of the volume of LIPA and sedentary time: a study which uses Zio may expect to estimate one-hour higher daily VLIPA and LIPA (95% CI = 0.83, 1.07 h/day) than a study which uses the waking-wear protocol.

### Generalizability, strengths, and limitations

Many studies estimate accelerometer cut points for older adults by enrolling moderate-sized groups to perform various activities (e.g., walking, sitting) in supervised settings [6, 18–21]. Accelerometry and calorimetry are assessed continuously, and the intensity of an activity is operationalized as its associated energy expenditure (e.g., sedentary may be defined as < 1.5 METs). Accelerometry cut points are estimated and evaluated by comparing accelerometry- and calorimetry-derived classifications of intensity. This type of study allows for the estimation of accuracy. Our study design precludes estimation of accuracy in comparison to a calorimetry gold-standard, but future assessment of our cut points via calorimetry could be devised. Additionally, our design has comparative benefits relative to a calorimetry-based design alone. First, our design utilizes a large, clinically diverse group of older adults to perform our mapping estimation, whereas calorimetry designs often focus on healthy individuals or narrower clinical groups. Second, since participants were observed over longer time horizons during free-living (3–6 days), and since our estimation focused on the harmonization of aggregate summaries, we have ensured that results will be reasonably comparable across studies and devices. When cut points are estimated and assessed at the minute level, small biases induced by the experimental design can compound when aggregating activity intensity across multiple days of data and potentially alter study conclusions [22].

Our new cut points should only be used with data from the Zio XT when activity intensity is operationalized as one-minute Mean Amplitude Deviation (MAD) among older adults. To facilitate researchers who wish to use other VMC cut-points for older adults wearing a waist-mounted ActiGraph GT3X, we provide a mapping which translates values of VMC to equivalent values of MAD from the Zio (Fig. 1, Appendix C). We note that Evenson et al. (2015) also provide cut-points for a waist-mounted GT3X which uses a low-frequency extension filter to calculate VMC [6], but because ARIC did not use this low-frequency extension filter, these cut-points would not be appropriate to map with our equations. Similarly, it would not be appropriate to use our mapping with VMC cut points derived for a younger population, body locations other than the waist (e.g., wrist), or other metrics of activity intensity than VMC (e.g., activity counts from the y-axis only).

The VMC cut-points originally adapted for ARIC were developed specifically for older women (60 to 91 years) [6, 7]. Since older men may generate different intensity of movement for a given energy expenditure, separate VMC cut points may be needed for older men for waist-worn devices, but the literature is mixed [18, 21]. Furthermore, even when we used the same cut points for both sexes, Zio characterized sex-specific differences in SST, LIPA, and MVPA differently than the GT3X (Fig. 4). For example, GT3X results indicated that women performed more light activity relative to men, but Zio results indicated that men and women performed similar amounts of light activity. We recommend that studies using our Zio cut points adjust for sex in their analyses but acknowledge that any significant sex-specific differences may reflect differences in movement, but underlying differences in energy expenditure remain unclear.

## Conclusions

The development of these cut-points provides an avenue for generating interpretable summaries of PA from Zio accelerometer data. Daily hours spent in activity intensity categories have been demonstrated to be important predictors of mortality in other studies which used other research-grade devices [23–25]. Future work should examine whether these associations can be replicated with studies which used Zio. Furthermore, because Zio is primarily used as an ambulatory ECG monitor, the ability to characterize PA intensity at each minute of the day could provide important contextual information for the analysis of the ECG waveforms. Future work may look at associations between activity intensity summaries and arrythmia burden.

## Supplementary Material

Supplemental material

## Figures and Tables

**Table 1:** Participant characteristics and comparison to entire ARIC Cohort at Visit 6

	Zio Study Participants (*n* = 381)		Other ARIC Participants (*n* = 3,622)		*P* *
	Median, N	(Q1, Q3, %)	Median, N	(Q1, Q3, %)	
**Age,** years	78	(75, 81)	79	(76, 83)	< 0.001
**Race/Ethnicity,** n(%)					
White	307	(80.6%)	2,707	(74.7%)	0.012
Non-White	74	(19.4%)	915	(25.3%)	
**Sex,** n (%)					
Male	165	(43.3%)	1472	(40.6%)	0.3
Female	216	(56.7%)	2150	(59.4%)	
**BMI,** kg/m^2^	27.2	(24.5, 30.8)	27.7	(24.5, 31.4)	0.15

* Fisher’s Exact tests were used to compare race and sex. Mann–Whitney tests were used for age and BMI

**Table 2:** Lower bound accelerometer cut-points to categorize physical activity intensity

Activity Category	GT3X VMC (60 s)^a^ [counts]	Percent of Total Wear Time	Zio XT MAD (60 s) [milli-g]	95% CI [milli-g]
Sedendary + Sleep	0	54.3%	0	
Very Light	76	28.4%	9.041	(8.867, 9.315)
Light	903	13.6%	28.187	(27.671, 28.758)
Moderate to Vigorous	2075	3.67%	58.083	(56.548, 59.690)

a Adaptation for ARIC used by Dooley et al. [6] based on Evenson et al. [5]

**Table 3:** Participant-level activity summaries by device using overlapping wear time (Scenario 1, *n* = 381)

	Sed. + Sleep (hrs/day)	Very Light (hrs/day)	Light (hrs/day)	Moderate - Vigorous	
				(hrs/day)	(min/week)
**Zio XT**, Only Data Overlapping with GT3X Waking Wear Protocol					
25th	16.29	3.27	1.37	0.19	78
Median	17.52	3.97	1.95	0.42	178
75th	18.8	4.75	2.46	0.71	296
**ActiGraph GT3X**, Waking Wear Protocol					
25th	16.5	3.28	1.41	0.19	81
Median	17.65	3.9	1.82	0.39	163
75th	18.73	4.74	2.43	0.67	280
**Pairwise Comparison**					
Pear. Cor	0.846	0.752	0.719	0.768	
ICC	0.846	0.752	0.719	0.769	
ICC 95% CI	(0.800, 0.880)	(0.689, 0.802)	(0.652, 0.770)	(0.684, 0.860)	
Mean Diff	−0.015	0.015	< 0.001	0.001	0.3
Mean Diff. 95% CI	(−0.116, 0.059)	(−0.065, 0.095)	(−0.060, 0.059)	(−0.032, 0.033)	(−13.4, 14.0)
SD Diff	1.002	0.791	0.592	0.324	136.1

**Table 4:** Participant-level activity summaries by device using device-specific wear time (Scenario 2, *n* = 378)

	Sed. + Sleep (hrs/day)	Very Light (hrs/day)	Light (hrs/day)	Moderate - Vigorous	
				(hrs/day)	(min/week)
**Zio XT**, 24-Hour Wear Protocol					
25th	15.2	3.94	1.49	0.19	79
Median	16.58	4.78	2.09	0.43	182
75th	17.86	5.67	2.7	0.73	307
**ActiGraph GT3X**, Waking Wear Protocol					
25th	16.36	3.29	1.41	0.19	80
Median	17.65	3.94	1.83	0.39	163
75th	18.73	4.77	2.47	0.67	280
**Pairwise Comparison**					
Pear. Cor	0.809	0.714	0.699	0.764	
ICC	0.700	0.551	0.677	0.765	
ICC 95% CI	(0.635, 0.756)	(0.472, 0.627)	(0.610, 0.737)	(0.688, 0.855)	
Mean Diff	−0.951	0.768	0.171	0.012	4.9
Mean Diff 95% CI	(−1.068, −0.834)	(0.677, 0.859)	(0.106, 0.237)	(−0.022, 0.045)	(−9.3, 19.0)
SD Diff	1.497	1.179	0.669	0.333	139.9

## Data Availability

ARIC is an ongoing cohort study from which data cannot be made available to the public. To obtain data from ARIC, a written request can be sent to the ARIC Data Coordinating Center. Further instructions may be found at https://aric.cscc.unc.edu/aric9/researchers/Obtain_Submit_Data.
