# Supplementary material for: Physical activity cut-points for older adults using the Zio XT onboard accelerometer
Source: BMC Digit Health. Author manuscript; Available in PMC 2025 Jul 31. (PMC12312660; doi:10.1186/s44247-024-00087-8)
Supplement: Supplemental material [file NIHMS2035206-supplement-Supplemental_material.docx]

**This version of the article has been accepted for publication, after peer review (when applicable) but is not the Version of Record and does not reflect post-acceptance improvements, or any corrections. The Version of Record is available online at: http://dx.doi.org/10.1186/s44247-024-00087-8.**

**Appendix**

**Activity Cut-Points for Older Adults using the Zio XT Patch Accelerometer**

Lacey H Etzkorn, Anis Davoudi, Erin E Dooley, Kelley P Gabriel, Lin Yee Chen, Ciprian M Crainiceanu, Jennifer A Schrack, Amal A Wanigatunga

**Corresponding Author:**

Lacey Etzkorn, Ph.D.

Postdoctoral Fellow

Center on Aging and Health and Department of Epidemiology

Johns Hopkins Bloomberg School of Public Health

Email: [letzkor1@jhu.edu](mailto:letzkor1@jhu.edu)

**Contents**

**Appendix A:** Zio XT and ActiGraph GT3X Data Examples

**Appendix B:** Activity Summary Measures

**Appendix C:** Tabulation of the Resulting QQ Mapping from Figure 1

**Appendix D:** Comparison of Zio Activity Summaries between Scenarios 1 and 2

**Appendix A:** Zio XT and ActiGraph GT3X Data Examples

Figure A1: Participant with Two Periods of Non-Wear


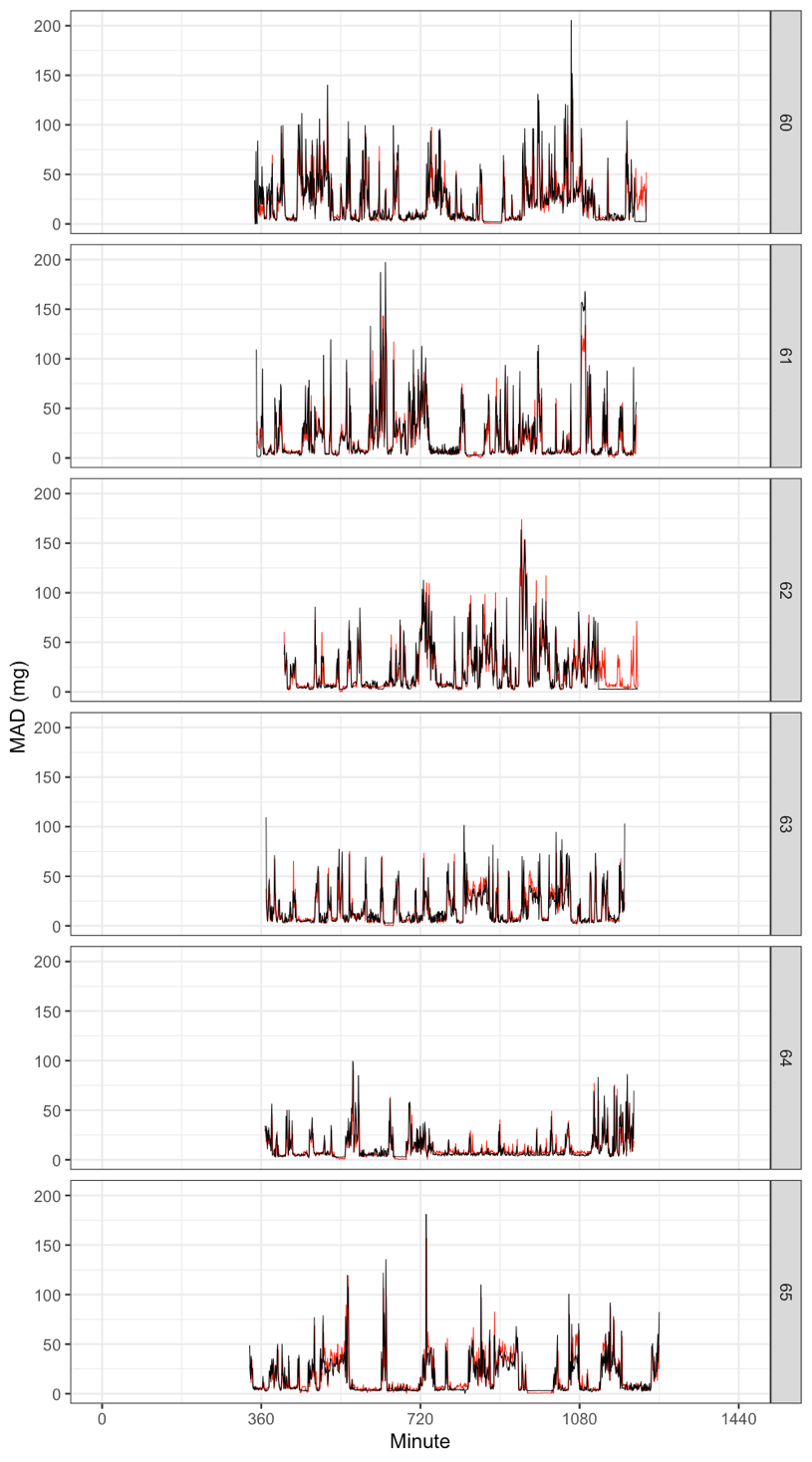


The timeseries for Zio XT is displayed in red, and the timeseries for Actigraph GT3X is displayed in black. Note, at the end of the wear days marked “60” and “62”, the black line is flat and near zero while the red line displays substantial activity. There is good agreement between the timeseries otherwise.

Figure A2: Excluded Participant with Highly Discordant MAD (Red) and VMC (Black) Timeseries


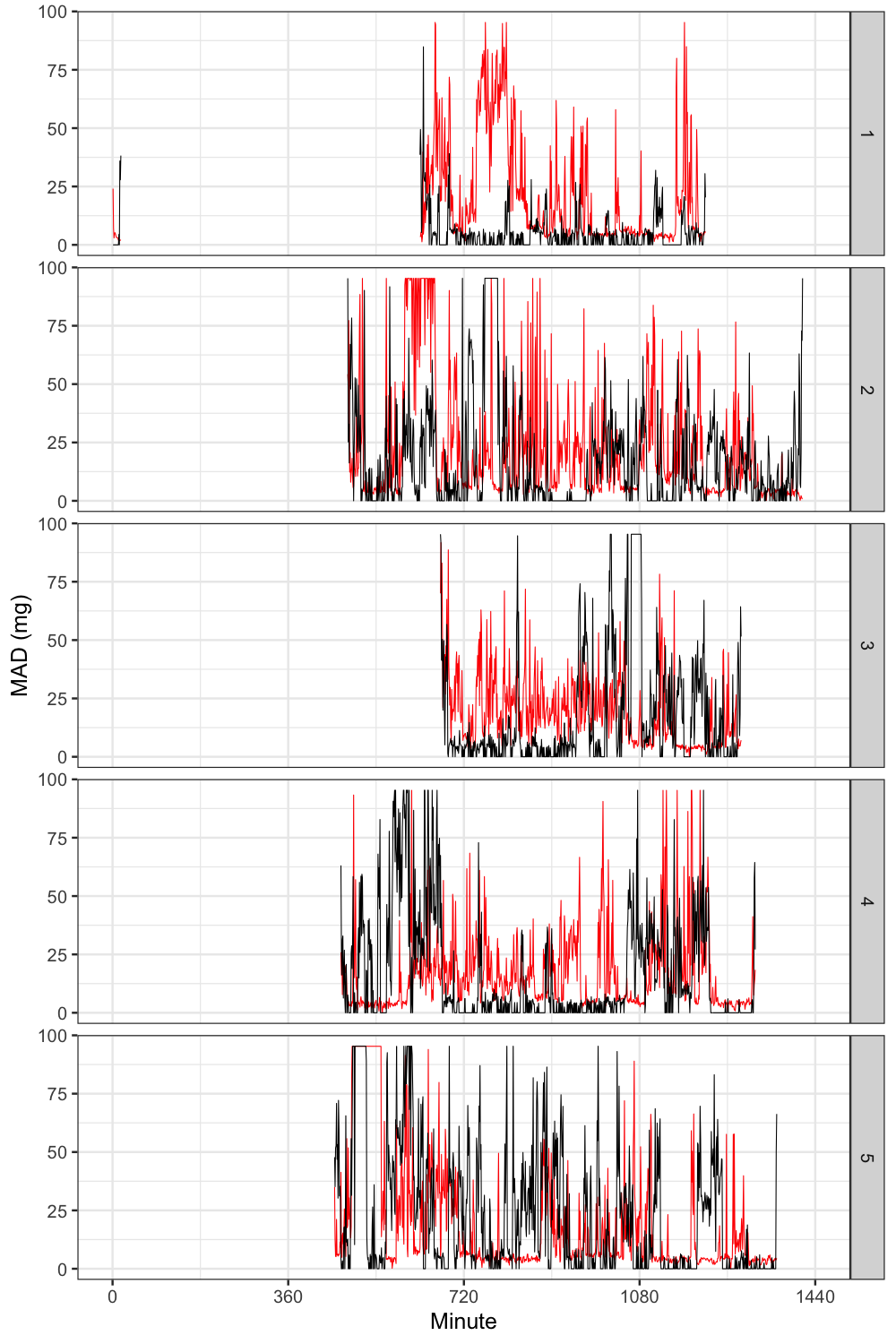


Here, the participant’s VMC timeseries were mapped to MAD using an earlier version of the mapping given in Appendix C.

**Appendix B: Activity Summary Measures: Mathematical Definitions**

**Minute-Level Mean Absolute Deviation (MAD)**

Let $x_{ij}\left( t \right),y_{ij}\left( t \right), z_{ij}\left( t \right)$ denote the triaxial accelerometer measurements at time of day $t\in(0,1440]$ on day *j* for person *i*. The mean absolute deviation at minute *k* = 0…1439 is defined as:

$$r_{ij}\left( t \right)=\sqrt{x_{ij}^{2}\left( t \right)+y_{ij}^{2}\left( t \right)+z_{ij}^{2}\left( t \right)}$$

$${MAD}_{ijk}=\frac{\sum_{k-1\leq t\leq k} |r_{ij}\left( t \right)-\bar{r}_{ijk}|}{\left| \left\{ t:k-1\leq t\leq k \right\} \right|}$$

**Non-Wear**

Let $\mathrm{NWG}_{\mathrm{ijk}}^{0}=1$ if person *i* on day *j* at minute *k* was not wearing the GT3X according to the Choi^14^ algorithm. Let $\mathrm{NWZ}_{\mathrm{ijk}}^{0}=1$ if person *i* on day *j* at minute *k* was not wearing the Zio according to the algorithm proposed by Etzkorn et al. (202X).

For scenario 1 (see section 2.4) non-wear is defined as non-wear from either device:

$N{WZ}_{ijk}=N{WG}_{ijk}=1-\left( 1-NWG_{ijk}^{0} \right)\cdot(1-NWZ_{ijk}^{0})$)

For scenario 2, non-wear is defined for each device separately:

$$N{WZ}_{ijk}= NWZ_{ijk}^{0}, N{WG}_{ijk}=NWG_{ijk}^{0}.$$

**Daily time in activity categories (SST, VLIPA, LIPA, MVPA), Scenario 1**

Let $\alpha_{G1}, \alpha_{G2},\alpha_{G3}, \alpha_{Z1}, \alpha_{Z2},\alpha_{Z3}$ denote the cut-points between consecutive activity categories for the GT3X and Zio respectively (See Table 1).

Daily Minutes of Sleep and Sedentary Time

$${SST}_{Zi}=\sum_{k=0}^{1439} \frac{1}{J_{ik}}\sum_{j=1}^{J_{ik}} \mathbb{I}\left\{ {MAD}_{ijk}<\alpha_{Z1} \right\}\cdot\left( 1-N{WZ}_{ijk} \right)+ N{WZ}_{ijk}$$

$$SST_{Gi}=\sum_{k=0}^{1439} \frac{1}{J_{ik}}\sum_{j=1}^{J_{ik}} \mathbb{I}\left\{ {VMC}_{ijk}<\alpha_{G1} \right\}\cdot\left( 1-N{WG}_{ijk} \right)+N{WG}_{ijk}$$

Daily Minutes of Very Light Intensity Activity

$$VLIPA_{Zi}=\sum_{k=0}^{1439} \frac{1}{J_{ik}}\sum_{j=1}^{J_{ik}} \mathbb{I}\left\{ \alpha_{Z1}\leq{MAD}_{ijk}<\alpha_{Z2} \right\}\cdot(1-N{WZ}_{ijk}), VLIPA_{Gi}=\sum_{k=0}^{1439} \frac{1}{J_{ik}}\sum_{j=1}^{J_{ik}} \mathbb{I}\left\{ \alpha_{G1}\leq{VMC}_{ijk}<\alpha_{G2} \right\}\cdot(1-N{WG}_{ijk})$$

Daily Minutes of Light Intensity Activity

$$LIPA_{Zi}=\sum_{k=0}^{1439} \frac{1}{J_{ik}}\sum_{j=1}^{J_{ik}} \mathbb{I}\left\{ \alpha_{Z2}\leq{MAD}_{ijk}<\alpha_{Z3} \right\}\cdot(1-N{WZ}_{ijk}), LIPA_{Gi}=\sum_{k=0}^{1439} \frac{1}{J_{ik}}\sum_{j=1}^{J_{ik}} \mathbb{I}\left\{ \alpha_{G2}\leq{VMC}_{ijk}<\alpha_{G3} \right\}\cdot(1-N{WG}_{ijk})$$

Daily Minutes of Moderate to Vigorous Physical Activity

$${MVPA}_{Zi}=\sum_{k=0}^{1439} \frac{1}{J_{ik}}\sum_{j=1}^{J_{ik}} \mathbb{I}\left\{ \alpha_{Z3}\leq{MAD}_{ijk} \right\}\cdot(1-N{WZ}_{ijk}), {MVPA}_{Gi}=\sum_{k=0}^{1439} \frac{1}{J_{ik}}\sum_{j=1}^{J_{ik}} \mathbb{I}\left\{ \alpha_{G3}\leq{VMC}_{ijk} \right\}\cdot(1-N{WG}_{ijk})$$

**Appendix C:** Tabulation of the Resulting QQ Mapping from Figure 1

| **VMC_G_** | **MAD_Z_** | **p (%)** | **VMC** | **MAD_Z_** | **p(%)** | **VMC** | **MAD_Z_** | **p(%)** | **VMC** | **MAD_Z_** | **p(%)** |
| --- | --- | --- | --- | --- | --- | --- | --- | --- | --- | --- | --- |
| 0 | 5.93 | 36.98 | 170 | 12.419 | 61.73 | 420 | 18.626 | 71.08 | 670 | 23.605 | 77.58 |
| 1 | 5.957 | 37.24 | 175 | 12.579 | 62.01 | 425 | 18.731 | 71.22 | 675 | 23.701 | 77.71 |
| 2 | 6.002 | 37.67 | 180 | 12.731 | 62.26 | 430 | 18.838 | 71.36 | 680 | 23.807 | 77.83 |
| 3 | 6.042 | 38.05 | 185 | 12.887 | 62.52 | 435 | 18.94 | 71.5 | 685 | 23.902 | 77.95 |
| 4 | 6.087 | 38.46 | 190 | 13.04 | 62.78 | 440 | 19.045 | 71.65 | 690 | 24.01 | 78.07 |
| 5 | 6.136 | 38.91 | 195 | 13.19 | 63.03 | 445 | 19.147 | 71.79 | 695 | 24.111 | 78.19 |
| 6 | 6.2 | 39.48 | 200 | 13.339 | 63.27 | 450 | 19.248 | 71.92 | 700 | 24.213 | 78.31 |
| 7 | 6.25 | 39.91 | 205 | 13.484 | 63.51 | 455 | 19.349 | 72.06 | 705 | 24.307 | 78.42 |
| 8 | 6.3 | 40.34 | 210 | 13.63 | 63.74 | 460 | 19.455 | 72.2 | 710 | 24.4 | 78.53 |
| 9 | 6.353 | 40.78 | 215 | 13.771 | 63.97 | 465 | 19.55 | 72.34 | 715 | 24.5 | 78.65 |
| 10 | 6.412 | 41.26 | 220 | 13.91 | 64.2 | 470 | 19.652 | 72.47 | 720 | 24.602 | 78.77 |
| 11 | 6.457 | 41.63 | 225 | 14.046 | 64.41 | 475 | 19.753 | 72.61 | 725 | 24.692 | 78.88 |
| 12 | 6.505 | 42 | 230 | 14.182 | 64.63 | 480 | 19.857 | 72.75 | 730 | 24.791 | 78.99 |
| 13 | 6.554 | 42.37 | 235 | 14.322 | 64.84 | 485 | 19.957 | 72.89 | 735 | 24.886 | 79.11 |
| 14 | 6.604 | 42.74 | 240 | 14.453 | 65.05 | 490 | 20.055 | 73.02 | 740 | 24.983 | 79.22 |
| 15 | 6.653 | 43.09 | 245 | 14.586 | 65.25 | 495 | 20.159 | 73.16 | 745 | 25.077 | 79.33 |
| 16 | 6.697 | 43.4 | 250 | 14.713 | 65.45 | 500 | 20.259 | 73.29 | 750 | 25.173 | 79.44 |
| 17 | 6.741 | 43.71 | 255 | 14.851 | 65.64 | 505 | 20.365 | 73.43 | 755 | 25.274 | 79.56 |
| 18 | 6.787 | 44.02 | 260 | 14.978 | 65.84 | 510 | 20.468 | 73.57 | 760 | 25.378 | 79.67 |
| 19 | 6.833 | 44.33 | 265 | 15.102 | 66.02 | 515 | 20.565 | 73.69 | 765 | 25.477 | 79.79 |
| 20 | 6.878 | 44.63 | 270 | 15.223 | 66.21 | 520 | 20.666 | 73.83 | 770 | 25.576 | 79.9 |
| 25 | 7.09 | 45.96 | 275 | 15.352 | 66.4 | 525 | 20.765 | 73.96 | 775 | 25.67 | 80.01 |
| 30 | 7.292 | 47.13 | 280 | 15.479 | 66.59 | 530 | 20.859 | 74.08 | 780 | 25.773 | 80.12 |
| 35 | 7.495 | 48.22 | 285 | 15.608 | 66.77 | 535 | 20.957 | 74.22 | 785 | 25.874 | 80.23 |
| 40 | 7.689 | 49.19 | 290 | 15.728 | 66.95 | 540 | 21.053 | 74.35 | 790 | 25.975 | 80.34 |
| 45 | 7.884 | 50.09 | 295 | 15.853 | 67.13 | 545 | 21.149 | 74.47 | 795 | 26.076 | 80.46 |
| 50 | 8.075 | 50.9 | 300 | 15.971 | 67.3 | 550 | 21.247 | 74.6 | 800 | 26.175 | 80.57 |
| 55 | 8.271 | 51.66 | 305 | 16.093 | 67.47 | 555 | 21.342 | 74.72 | 805 | 26.274 | 80.68 |
| 60 | 8.463 | 52.37 | 310 | 16.213 | 67.65 | 560 | 21.446 | 74.86 | 810 | 26.372 | 80.79 |
| 65 | 8.662 | 53.04 | 315 | 16.334 | 67.82 | 565 | 21.544 | 74.99 | 815 | 26.471 | 80.89 |
| 70 | 8.854 | 53.67 | 320 | 16.448 | 67.99 | 570 | 21.637 | 75.11 | 820 | 26.576 | 81 |
| 75 | 9.041 | 54.26 | 325 | 16.563 | 68.16 | 575 | 21.739 | 75.24 | 825 | 26.676 | 81.11 |
| 80 | 9.232 | 54.82 | 330 | 16.684 | 68.33 | 580 | 21.834 | 75.36 | 830 | 26.774 | 81.22 |
| 85 | 9.422 | 55.35 | 335 | 16.793 | 68.49 | 585 | 21.934 | 75.49 | 835 | 26.872 | 81.33 |
| 90 | 9.616 | 55.86 | 340 | 16.91 | 68.66 | 590 | 22.033 | 75.62 | 840 | 26.965 | 81.42 |
| 95 | 9.806 | 56.35 | 345 | 17.018 | 68.82 | 595 | 22.128 | 75.74 | 845 | 27.063 | 81.53 |
| 100 | 9.99 | 56.82 | 350 | 17.126 | 68.97 | 600 | 22.224 | 75.86 | 850 | 27.167 | 81.64 |
| 105 | 10.174 | 57.26 | 355 | 17.238 | 69.14 | 605 | 22.326 | 75.99 | 855 | 27.267 | 81.74 |
| 110 | 10.359 | 57.68 | 360 | 17.347 | 69.29 | 610 | 22.429 | 76.12 | 860 | 27.363 | 81.84 |
| 115 | 10.538 | 58.08 | 365 | 17.457 | 69.44 | 615 | 22.529 | 76.25 | 865 | 27.455 | 81.94 |
| 120 | 10.715 | 58.47 | 370 | 17.569 | 69.6 | 620 | 22.628 | 76.37 | 870 | 27.55 | 82.04 |
| 125 | 10.895 | 58.85 | 375 | 17.677 | 69.75 | 625 | 22.723 | 76.49 | 875 | 27.649 | 82.15 |
| 130 | 11.073 | 59.21 | 380 | 17.786 | 69.9 | 630 | 22.82 | 76.61 | 880 | 27.745 | 82.25 |
| 135 | 11.251 | 59.56 | 385 | 17.891 | 70.05 | 635 | 22.922 | 76.74 | 885 | 27.841 | 82.35 |
| 140 | 11.428 | 59.91 | 390 | 17.991 | 70.2 | 640 | 23.02 | 76.87 | 890 | 27.944 | 82.45 |
| 145 | 11.6 | 60.24 | 395 | 18.1 | 70.35 | 645 | 23.114 | 76.99 | 895 | 28.046 | 82.56 |
| 150 | 11.767 | 60.56 | 400 | 18.205 | 70.5 | 650 | 23.217 | 77.11 | 900 | 28.148 | 82.66 |
| 155 | 11.933 | 60.86 | 405 | 18.314 | 70.65 | 655 | 23.313 | 77.23 | 905 | 28.261 | 82.76 |
| 160 | 12.096 | 61.15 | 410 | 18.42 | 70.79 | 660 | 23.41 | 77.35 | 910 | 28.357 | 82.86 |
| 165 | 12.256 | 61.44 | 415 | 18.522 | 70.93 | 665 | 23.509 | 77.47 | 915 | 28.448 | 82.96 |

**Appendix C:** Tabulation of the Resulting QQ Mapping from Figure 1 (CONTINUED)

| **VMC** | **MAD_Z_** | **p (%)** | **VMC** | **MAD_Z_** | **p(%)** | **VMC** | **MAD_Z_** | **p(%)** | **VMC** | **MAD_Z_** | **p(%)** |
| --- | --- | --- | --- | --- | --- | --- | --- | --- | --- | --- | --- |
| 920 | 28.545 | 83.06 | 1170 | 33.761 | 87.55 | 1420 | 39.493 | 91.03 | 1670 | 45.938 | 93.6 |
| 925 | 28.651 | 83.16 | 1175 | 33.863 | 87.62 | 1425 | 39.612 | 91.09 | 1675 | 46.077 | 93.64 |
| 930 | 28.75 | 83.25 | 1180 | 33.969 | 87.7 | 1430 | 39.728 | 91.15 | 1680 | 46.217 | 93.68 |
| 935 | 28.857 | 83.35 | 1185 | 34.076 | 87.78 | 1435 | 39.852 | 91.2 | 1685 | 46.357 | 93.73 |
| 940 | 28.951 | 83.45 | 1190 | 34.188 | 87.85 | 1440 | 39.979 | 91.27 | 1690 | 46.495 | 93.77 |
| 945 | 29.051 | 83.55 | 1195 | 34.299 | 87.93 | 1445 | 40.105 | 91.32 | 1695 | 46.636 | 93.81 |
| 950 | 29.15 | 83.65 | 1200 | 34.405 | 88.01 | 1450 | 40.213 | 91.38 | 1700 | 46.772 | 93.86 |
| 955 | 29.251 | 83.75 | 1205 | 34.516 | 88.09 | 1455 | 40.34 | 91.44 | 1705 | 46.906 | 93.9 |
| 960 | 29.349 | 83.84 | 1210 | 34.624 | 88.16 | 1460 | 40.464 | 91.49 | 1710 | 47.044 | 93.94 |
| 965 | 29.458 | 83.95 | 1215 | 34.739 | 88.24 | 1465 | 40.594 | 91.55 | 1715 | 47.178 | 93.98 |
| 970 | 29.563 | 84.04 | 1220 | 34.855 | 88.31 | 1470 | 40.724 | 91.61 | 1720 | 47.32 | 94.02 |
| 975 | 29.668 | 84.14 | 1225 | 34.964 | 88.39 | 1475 | 40.84 | 91.66 | 1725 | 47.463 | 94.06 |
| 980 | 29.773 | 84.23 | 1230 | 35.068 | 88.46 | 1480 | 40.958 | 91.72 | 1730 | 47.604 | 94.1 |
| 985 | 29.871 | 84.33 | 1235 | 35.186 | 88.54 | 1485 | 41.081 | 91.77 | 1735 | 47.739 | 94.14 |
| 990 | 29.982 | 84.43 | 1240 | 35.294 | 88.61 | 1490 | 41.203 | 91.83 | 1740 | 47.882 | 94.18 |
| 995 | 30.086 | 84.52 | 1245 | 35.406 | 88.68 | 1495 | 41.331 | 91.88 | 1745 | 48.03 | 94.22 |
| 1000 | 30.185 | 84.61 | 1250 | 35.514 | 88.76 | 1500 | 41.456 | 91.93 | 1750 | 48.18 | 94.26 |
| 1005 | 30.287 | 84.71 | 1255 | 35.623 | 88.83 | 1505 | 41.57 | 91.99 | 1755 | 48.312 | 94.3 |
| 1010 | 30.387 | 84.8 | 1260 | 35.735 | 88.9 | 1510 | 41.705 | 92.04 | 1760 | 48.451 | 94.34 |
| 1015 | 30.486 | 84.89 | 1265 | 35.848 | 88.97 | 1515 | 41.838 | 92.1 | 1765 | 48.594 | 94.38 |
| 1020 | 30.584 | 84.98 | 1270 | 35.964 | 89.04 | 1520 | 41.979 | 92.16 | 1770 | 48.744 | 94.42 |
| 1025 | 30.689 | 85.08 | 1275 | 36.079 | 89.12 | 1525 | 42.108 | 92.21 | 1775 | 48.893 | 94.46 |
| 1030 | 30.793 | 85.17 | 1280 | 36.192 | 89.19 | 1530 | 42.234 | 92.27 | 1780 | 49.047 | 94.5 |
| 1035 | 30.9 | 85.26 | 1285 | 36.311 | 89.26 | 1535 | 42.36 | 92.32 | 1785 | 49.187 | 94.53 |
| 1040 | 31.002 | 85.35 | 1290 | 36.431 | 89.34 | 1540 | 42.488 | 92.37 | 1790 | 49.329 | 94.57 |
| 1045 | 31.105 | 85.44 | 1295 | 36.54 | 89.4 | 1545 | 42.606 | 92.42 | 1795 | 49.467 | 94.61 |
| 1050 | 31.208 | 85.53 | 1300 | 36.656 | 89.47 | 1550 | 42.741 | 92.47 | 1800 | 49.603 | 94.64 |
| 1055 | 31.313 | 85.62 | 1305 | 36.77 | 89.54 | 1555 | 42.867 | 92.52 | 1805 | 49.748 | 94.68 |
| 1060 | 31.425 | 85.71 | 1310 | 36.881 | 89.61 | 1560 | 42.992 | 92.57 | 1810 | 49.894 | 94.72 |
| 1065 | 31.531 | 85.8 | 1315 | 37.001 | 89.68 | 1565 | 43.122 | 92.62 | 1815 | 50.037 | 94.75 |
| 1070 | 31.633 | 85.88 | 1320 | 37.122 | 89.75 | 1570 | 43.246 | 92.67 | 1820 | 50.173 | 94.79 |
| 1075 | 31.735 | 85.97 | 1325 | 37.247 | 89.82 | 1575 | 43.38 | 92.72 | 1825 | 50.332 | 94.82 |
| 1080 | 31.841 | 86.06 | 1330 | 37.362 | 89.89 | 1580 | 43.514 | 92.76 | 1830 | 50.477 | 94.86 |
| 1085 | 31.937 | 86.14 | 1335 | 37.474 | 89.95 | 1585 | 43.646 | 92.81 | 1835 | 50.62 | 94.89 |
| 1090 | 32.041 | 86.23 | 1340 | 37.588 | 90.02 | 1590 | 43.773 | 92.86 | 1840 | 50.769 | 94.93 |
| 1095 | 32.147 | 86.31 | 1345 | 37.702 | 90.08 | 1595 | 43.9 | 92.91 | 1845 | 50.922 | 94.96 |
| 1100 | 32.254 | 86.4 | 1350 | 37.826 | 90.15 | 1600 | 44.031 | 92.95 | 1850 | 51.07 | 95 |
| 1105 | 32.363 | 86.49 | 1355 | 37.94 | 90.22 | 1605 | 44.164 | 93 | 1855 | 51.216 | 95.03 |
| 1110 | 32.467 | 86.57 | 1360 | 38.053 | 90.28 | 1610 | 44.3 | 93.05 | 1860 | 51.361 | 95.07 |
| 1115 | 32.565 | 86.65 | 1365 | 38.168 | 90.34 | 1615 | 44.434 | 93.1 | 1865 | 51.524 | 95.1 |
| 1120 | 32.679 | 86.74 | 1370 | 38.278 | 90.4 | 1620 | 44.572 | 93.15 | 1870 | 51.67 | 95.14 |
| 1125 | 32.787 | 86.82 | 1375 | 38.395 | 90.46 | 1625 | 44.709 | 93.19 | 1875 | 51.818 | 95.17 |
| 1130 | 32.892 | 86.9 | 1380 | 38.51 | 90.53 | 1630 | 44.859 | 93.24 | 1880 | 51.97 | 95.21 |
| 1135 | 32.999 | 86.98 | 1385 | 38.627 | 90.59 | 1635 | 44.998 | 93.29 | 1885 | 52.117 | 95.24 |
| 1140 | 33.112 | 87.07 | 1390 | 38.747 | 90.65 | 1640 | 45.13 | 93.33 | 1890 | 52.273 | 95.27 |
| 1145 | 33.22 | 87.15 | 1395 | 38.867 | 90.71 | 1645 | 45.262 | 93.38 | 1895 | 52.431 | 95.31 |
| 1150 | 33.326 | 87.23 | 1400 | 38.991 | 90.78 | 1650 | 45.403 | 93.42 | 1900 | 52.588 | 95.34 |
| 1155 | 33.435 | 87.31 | 1405 | 39.121 | 90.84 | 1655 | 45.534 | 93.47 | 1905 | 52.752 | 95.37 |
| 1160 | 33.549 | 87.39 | 1410 | 39.244 | 90.9 | 1660 | 45.669 | 93.51 | 1910 | 52.926 | 95.41 |
| 1165 | 33.653 | 87.47 | 1415 | 39.366 | 90.96 | 1665 | 45.799 | 93.55 | 1915 | 53.077 | 95.44 |

**Appendix C:** Tabulation of the Resulting QQ Mapping from Figure 1 (CONTINUED)

| **VMC_G_** | **MAD_Z_** | **p(%)** | **VMC** | **MAD_Z_** | **p(%)** | **VMC** | **MAD_Z_** | **p(%)** | **VMC** | **MAD_Z_** | **p(%)** |
| --- | --- | --- | --- | --- | --- | --- | --- | --- | --- | --- | --- |
| 1920 | 53.226 | 95.47 | 2170 | 61.374 | 96.79 | 2420 | 70.605 | 97.71 | 2670 | 81.02 | 98.34 |
| 1925 | 53.381 | 95.5 | 2175 | 61.541 | 96.82 | 2425 | 70.814 | 97.73 | 2675 | 81.204 | 98.35 |
| 1930 | 53.536 | 95.53 | 2180 | 61.734 | 96.84 | 2430 | 71.023 | 97.74 | 2680 | 81.42 | 98.36 |
| 1935 | 53.682 | 95.56 | 2185 | 61.925 | 96.86 | 2435 | 71.241 | 97.76 | 2685 | 81.672 | 98.37 |
| 1940 | 53.819 | 95.59 | 2190 | 62.097 | 96.88 | 2440 | 71.434 | 97.77 | 2690 | 81.897 | 98.38 |
| 1945 | 53.957 | 95.62 | 2195 | 62.259 | 96.9 | 2445 | 71.649 | 97.79 | 2695 | 82.113 | 98.39 |
| 1950 | 54.123 | 95.65 | 2200 | 62.434 | 96.92 | 2450 | 71.864 | 97.8 | 2700 | 82.367 | 98.4 |
| 1955 | 54.286 | 95.68 | 2205 | 62.628 | 96.94 | 2455 | 72.05 | 97.82 | 2705 | 82.569 | 98.41 |
| 1960 | 54.447 | 95.71 | 2210 | 62.796 | 96.96 | 2460 | 72.238 | 97.83 | 2710 | 82.804 | 98.42 |
| 1965 | 54.607 | 95.75 | 2215 | 62.967 | 96.98 | 2465 | 72.465 | 97.85 | 2715 | 83.054 | 98.43 |
| 1970 | 54.773 | 95.78 | 2220 | 63.176 | 97 | 2470 | 72.655 | 97.86 | 2720 | 83.256 | 98.44 |
| 1975 | 54.912 | 95.8 | 2225 | 63.357 | 97.02 | 2475 | 72.866 | 97.88 | 2725 | 83.488 | 98.45 |
| 1980 | 55.083 | 95.83 | 2230 | 63.558 | 97.05 | 2480 | 73.082 | 97.89 | 2730 | 83.728 | 98.46 |
| 1985 | 55.221 | 95.86 | 2235 | 63.746 | 97.07 | 2485 | 73.29 | 97.91 | 2735 | 83.956 | 98.47 |
| 1990 | 55.371 | 95.89 | 2240 | 63.891 | 97.08 | 2490 | 73.502 | 97.92 | 2740 | 84.199 | 98.48 |
| 1995 | 55.525 | 95.91 | 2245 | 64.057 | 97.1 | 2495 | 73.719 | 97.94 | 2745 | 84.409 | 98.49 |
| 2000 | 55.671 | 95.94 | 2250 | 64.222 | 97.12 | 2500 | 73.934 | 97.95 | 2750 | 84.634 | 98.5 |
| 2005 | 55.83 | 95.97 | 2255 | 64.394 | 97.14 | 2505 | 74.137 | 97.96 | 2755 | 84.843 | 98.51 |
| 2010 | 55.981 | 96 | 2260 | 64.542 | 97.16 | 2510 | 74.368 | 97.97 | 2760 | 85.06 | 98.52 |
| 2015 | 56.135 | 96.02 | 2265 | 64.731 | 97.18 | 2515 | 74.567 | 97.99 | 2765 | 85.281 | 98.53 |
| 2020 | 56.294 | 96.05 | 2270 | 64.916 | 97.19 | 2520 | 74.759 | 98 | 2770 | 85.475 | 98.54 |
| 2025 | 56.455 | 96.08 | 2275 | 65.126 | 97.21 | 2525 | 74.996 | 98.01 | 2775 | 85.67 | 98.55 |
| 2030 | 56.612 | 96.11 | 2280 | 65.325 | 97.23 | 2530 | 75.201 | 98.03 | 2780 | 85.868 | 98.55 |
| 2035 | 56.762 | 96.13 | 2285 | 65.532 | 97.25 | 2535 | 75.397 | 98.04 | 2785 | 86.126 | 98.56 |
| 2040 | 56.943 | 96.16 | 2290 | 65.712 | 97.27 | 2540 | 75.587 | 98.05 | 2790 | 86.415 | 98.57 |
| 2045 | 57.103 | 96.19 | 2295 | 65.923 | 97.29 | 2545 | 75.8 | 98.06 | 2795 | 86.653 | 98.58 |
| 2050 | 57.286 | 96.21 | 2300 | 66.118 | 97.31 | 2550 | 76.008 | 98.07 | 2800 | 86.859 | 98.59 |
| 2055 | 57.451 | 96.24 | 2305 | 66.309 | 97.33 | 2555 | 76.215 | 98.09 | 2805 | 87.132 | 98.6 |
| 2060 | 57.617 | 96.26 | 2310 | 66.485 | 97.35 | 2560 | 76.416 | 98.1 | 2810 | 87.387 | 98.61 |
| 2065 | 57.775 | 96.29 | 2315 | 66.646 | 97.37 | 2565 | 76.607 | 98.11 | 2815 | 87.646 | 98.62 |
| 2070 | 57.948 | 96.31 | 2320 | 66.837 | 97.38 | 2570 | 76.795 | 98.12 | 2820 | 87.88 | 98.63 |
| 2075 | 58.116 | 96.34 | 2325 | 67.016 | 97.4 | 2575 | 77.018 | 98.13 | 2825 | 88.129 | 98.64 |
| 2080 | 58.281 | 96.36 | 2330 | 67.173 | 97.42 | 2580 | 77.23 | 98.15 | 2830 | 88.313 | 98.64 |
| 2085 | 58.438 | 96.39 | 2335 | 67.343 | 97.43 | 2585 | 77.431 | 98.16 | 2835 | 88.564 | 98.65 |
| 2090 | 58.595 | 96.41 | 2340 | 67.55 | 97.45 | 2590 | 77.671 | 98.17 | 2840 | 88.82 | 98.66 |
| 2095 | 58.759 | 96.44 | 2345 | 67.75 | 97.47 | 2595 | 77.87 | 98.18 | 2845 | 89.076 | 98.67 |
| 2100 | 58.94 | 96.46 | 2350 | 67.934 | 97.49 | 2600 | 78.077 | 98.19 | 2850 | 89.352 | 98.68 |
| 2105 | 59.105 | 96.49 | 2355 | 68.14 | 97.5 | 2605 | 78.239 | 98.2 | 2855 | 89.584 | 98.69 |
| 2110 | 59.282 | 96.51 | 2360 | 68.288 | 97.52 | 2610 | 78.501 | 98.22 | 2860 | 89.815 | 98.7 |
| 2115 | 59.441 | 96.53 | 2365 | 68.478 | 97.54 | 2615 | 78.665 | 98.23 | 2865 | 90.065 | 98.7 |
| 2120 | 59.623 | 96.56 | 2370 | 68.659 | 97.55 | 2620 | 78.906 | 98.24 | 2870 | 90.263 | 98.71 |
| 2125 | 59.774 | 96.58 | 2375 | 68.882 | 97.57 | 2625 | 79.106 | 98.25 | 2875 | 90.493 | 98.72 |
| 2130 | 59.951 | 96.61 | 2380 | 69.087 | 97.59 | 2630 | 79.308 | 98.26 | 2880 | 90.771 | 98.73 |
| 2135 | 60.141 | 96.63 | 2385 | 69.281 | 97.6 | 2635 | 79.549 | 98.27 | 2885 | 90.958 | 98.73 |
| 2140 | 60.306 | 96.66 | 2390 | 69.46 | 97.62 | 2640 | 79.796 | 98.28 | 2890 | 91.167 | 98.74 |
| 2145 | 60.484 | 96.68 | 2395 | 69.658 | 97.63 | 2645 | 79.998 | 98.29 | 2895 | 91.45 | 98.75 |
| 2150 | 60.669 | 96.7 | 2400 | 69.862 | 97.65 | 2650 | 80.193 | 98.3 | 2900 | 91.686 | 98.76 |
| 2155 | 60.837 | 96.73 | 2405 | 70.039 | 97.66 | 2655 | 80.384 | 98.31 | 2905 | 91.87 | 98.76 |
| 2160 | 61.015 | 96.75 | 2410 | 70.197 | 97.68 | 2660 | 80.588 | 98.32 | 2910 | 92.065 | 98.77 |
| 2165 | 61.211 | 96.77 | 2415 | 70.397 | 97.69 | 2665 | 80.82 | 98.33 | 2915 | 92.361 | 98.78 |

**Appendix D: Comparison of Zio Activity Summaries between Scenarios 1 and 2**

|  | **Sed. + Sleep** (hrs/day) | **Very Light**  (hrs/day) | **Light**  (hrs/day) | **Moderate – Vigorous** | |
| --- | --- | --- | --- | --- | --- |
|  |  |  |  | (hrs/day) | **(**min/week) |
| **Zio XT**, 24-Hour Wear Protocol | | | | | |
| 25^th^ | 15.2 | 3.94 | 1.49 | 0.19 | 79 |
| Median | 16.58 | 4.78 | 2.09 | 0.43 | 182 |
| 75^th^ | 17.86 | 5.67 | 2.7 | 0.73 | 307 |
| **Zio XT**, Overlap with GT3X Waking Wear Protocol | | | | | |
| 25^th^ | 16.29 | 3.27 | 1.37 | 0.19 | 78 |
| Median | 17.52 | 3.97 | 1.95 | 0.42 | 178 |
| 75^th^ | 18.8 | 4.75 | 2.46 | 0.71 | 296 |
| **Pairwise Comparison** | | | | | |
| Pear. Cor. | 0.961 | 0.945 | 0.983 | 0.997 | |
| ICC | 0.830 | 0.744 | 0.954 | 0.996 | |
| ICC  95% CI | (0.796, 0.859) | (0.706, 0.782) | (0.941, 0.964) | (0.993, 0.998) | |
| Mean Diff. | 0.99 | -0.78 | -0.19 | -0.018 | -7.5 |
| Mean Diff.  95% CI | (0.94, 1.05) | (-0.82, -0.74) | (-0.21, -0.18) | (-0.022, -0.014) | (-9.3, -5.8) |
| SD Diff. | 1.12 | 0.88 | 0.26 | 0.045 | 18.8 |

**Figure D1: Discrepancies in Zio Summaries between Scenarios 1 and 2**

**
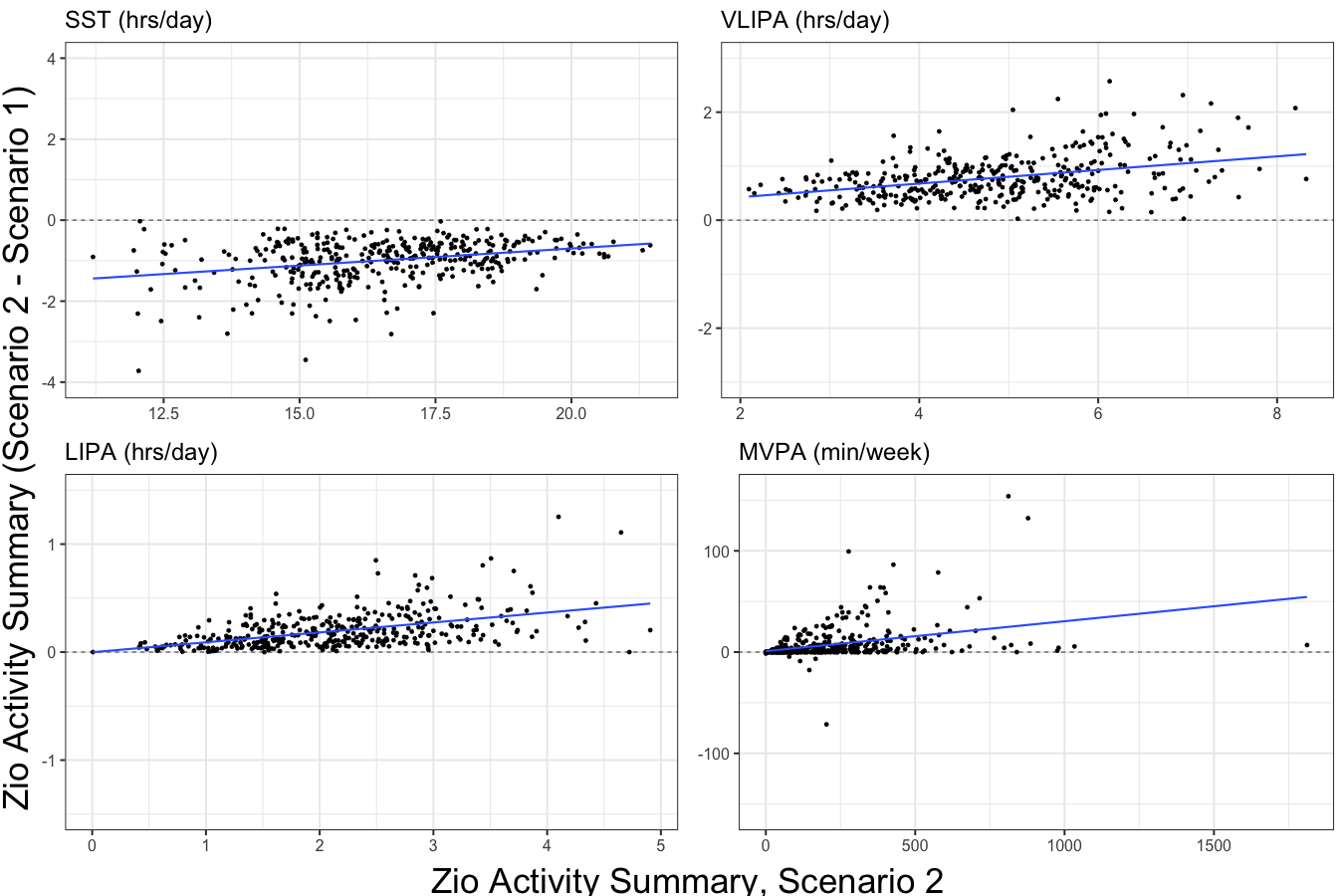
**

**Note:** SST, VLIPA, LIPA, and MVPA calculated in Scenario 2 use all 24 hours of Zio data. For Scenario 1, all time periods when the GT3X was not worn are classified as inactive time before other summaries are calculated.
